# Effects of anthropogenic noise on endocrine and reproductive function in White's treefrog, *Litoria caerulea*

**DOI:** 10.1093/conphys/cou061

**Published:** 2015-01-16

**Authors:** Kristine Kaiser, Julia Devito, Caitlin G. Jones, Adam Marentes, Rachel Perez, Lisa Umeh, Regina M. Weickum, Kathryn E. McGovern, Emma H. Wilson, Wendy Saltzman

**Affiliations:** 1 Department of Biology, University of California, Riverside, CA 92521, USA; 2 Division of Biomedical Sciences, School of Medicine, University of California, Riverside, CA 92521, USA

**Keywords:** Corticosterone, frog, sperm count, sperm viability, traffic noise

## Abstract

Urbanization is a major driver of ecological change and comes with a suite of habitat modifications, including alterations to the local temperature, precipitation, light and noise regimes. Although many recent studies have investigated the behavioural and ecological ramifications of urbanization, physiological work in this area has lagged. We tested the hypothesis that anthropogenic noise is a stressor for amphibians and that chronic exposure to such noise leads to reproductive suppression. In the laboratory, we exposed male White's treefrogs, *Litoria caerulea*, to conspecific chorus noise either alone or coupled with pre-recorded traffic noise nightly for 1 week. Frogs presented with anthropogenic noise had significantly higher circulating concentrations of corticosterone and significantly decreased sperm count and sperm viability than did control frogs. These results suggest that in addition to having behavioural and ecological effects, anthropogenic change might alter physiology and Darwinian fitness. Future work should integrate disparate fields such as behaviour, ecology and physiology to elucidate fully organisms’ responses to habitat change.

## Abstract (precis)

Urbanization brings the introduction of roads and anthropogenic noise. Noise has negative health outcomes across vertebrates. White's treefrogs (*Litoria caerulea*) exposed to ecologically relevant levels of traffic noise for one week had elevated circulating corticosterone levels and decreased sperm count and sperm viability relative to controls.

## Introduction

Urbanization is increasing globally at a rapid rate, with few places escaping human-induced change (Ellis, 2011). As a result, urbanization has become a major driver of ecological change around the world (Ellis and Ramankutty, 2008; Ellis, 2011; Fragkias *et al.*, 2013). Increased anthropogenic activity and its byproducts often lead to changes in a variety of habitat parameters, including microclimate, species distributions and densities and introduction of chemical contaminants (Goosem, 2000; Kowarik, 2011; Pickett *et al.*, 2011). Less-studied changes include the introduction of pollution, such as light and anthropogenic noise (e.g. traffic noise), especially at fine scales (Longcore and Rich, 2004; Brumm and Slabbekoorn, 2005; Sun and Narins, 2005; Kaiser and Hammers, 2009; Parris *et al.*, 2009; Kaiser *et al.*, 2011).

These types of habitat alterations can lead to stress in animals (reviewed by Bonier, 2012). Chronic stress has been linked to detrimental effects, including suppression of reproductive behaviour and physiology (Moore and Miller, 1984; Sapolsky *et al.*, 2000; Gore *et al.*, 2006; Wingfield and Sapolsky, 2003). In vertebrates, one marker of stress is increased secretion of the glucocorticoid hormones [i.e. cortisol and/or corticosterone (CORT)]. Although habitat change has been associated with increases in CORT across taxa (Newcomb-Homan *et al.*, 2003; Martínez-Mota *et al.*, 2007; Herrera *et al.*, 2009; Sheriff *et al.*, 2010), there remains a dearth of controlled studies investigating the impact of urbanization on endocrine function (reviewed by Bonier, 2012). Even fewer studies trace the downstream effects of such stressors (e.g. pathology) or their ramifications for fitness.

Despite widespread interest in the ecological implications of habitat change, few studies have examined the effect of habitat change on amphibian physiology (Newcomb-Homan *et al*., 2003; Skelly *et al.*, 2006). The role of acute stress in amphibian ecology has recently received attention (e.g. Gabor *et al.*, 2013; Narayan *et al.*, 2013; Narayan and Hero, 2013) yet little is known about the physiological and behavioural effects of chronic stress in amphibians or the role of chronic stress in population dynamics. In other taxa, however, stress hormones interact with other systems in complex ways, including suppression of hormones responsible for reproduction as well as direct effects on the gonads (Wingfield and Sapolsky, 2003).

We tested the hypothesis that noise contamination associated with urbanization—specifically, traffic noise—is stressful to amphibians and is detrimental to their reproductive function. Amphibians may be among the most susceptible vertebrates to habitat loss and change, in part because they are among the least vagile (Cushman, 2006), but also because they are constrained by both their natural history and their permeable skin in the types of matrix that they can traverse (Houlahan and Findlay, 2003). We chose traffic noise as a generic anthropogenic stressor for several reasons. First, noise increases circulating CORT concentrations in many vertebrates, including frogs (Barber *et al.*, 2010; Bonier, 2012; Tennessen *et al.*, 2014). Second, as urbanization increases, anthropogenic noise becomes ecologically relevant to more and more species. Finally, environmental noise has been linked to shifts in communication in frogs and birds (Narins *et al.*, 2004; Arch *et al.*, 2008), and traffic noise induces behavioural changes in many taxa, including frogs (Slabbekoorn and Ripmeester, 2008; Kaiser and Hammers, 2009; Barber *et al*., 2010; Kaiser *et al*., 2011; Tennessen *et al*., 2014).

We predicted that chronic exposure to pre-recorded anthropogenic noise at ecologically relevant levels would increase circulating CORT concentrations in treefrogs relative to control treefrogs presented with natural chorus noise alone, and that these same frogs would show decreases in sperm count and sperm viability. Exposure to natural chorus noise alone can increase secretion of CORT in frogs (Burmeister and Wilczynski, 2000), and we chose this control to allow for an ecologically relevant characterization of the CORT response to anthropogenic noise specifically. We thus expected that all frogs would demonstrate an increase in circulating levels of plasma CORT across the period of noise exposure, but that frogs presented with anthropogenic noise would show a greater increase in CORT levels as well as decreases in measures of reproductive function.

## Materials and methods

### Animals and experimental design

We used White's treefrog (*Litoria caerulea*), a frog broadly distributed across Australia and Papua New Guinea. Considered of least conservation concern (IUCN, 2013) and a member of the speciose and diverse family Hylidae, *L. caerulea* has been used widely in physiological and ecological studies (Buttemer, 1990; Baker and Waights, 1994; Coddington and Cree, 1995; Warburg *et al.*, 2000; Woodhams *et al.*, 2007; Voyles *et al.*, 2009). Due to restrictions on acquiring adult animals from their native range, we acquired 29 adult male frogs that were captive bred in California, USA for this experiment.

This study was conducted during September–October, near the end of the North American breeding season for this species. We confirmed that frogs were still in breeding condition by verifying the presence of nuptial pads on animals’ front feet (B. Mailloux, personal communication). Additionally, we used two separate control groups to control for possible seasonal effects (see below). Frogs were maintained in plastic tanks approximately 40 cm × 24 cm × 32 cm (size XL; Kritter Keepers, San Marcos, CA, USA) with dechlorinated tap water *ad libitum* and a 10 cm length of PVC pipe for enrichment. Frogs were fed crickets twice weekly; uneaten crickets were removed 24 h prior to blood collection. Lighting and temperature were controlled on a 12 h–12 h light–dark cycle (lights on at 09.00 h) and at 21–23°C, respectively. Animals were housed in the laboratory for at least 1 month prior to experiments. All procedures were approved by the University of California, Riverside Institutional Animal Care and Use Committee. The University of California, Riverside is fully accredited by the Association for Assessment and Accreditation of Laboratory Animal Care.

We compared plasma CORT concentrations, sperm count and sperm viability between experimental frogs, which were presented with recordings of conspecific choruses as well as anthropogenic noise, and control frogs, which were exposed only to conspecific playbacks. Animals were exposed to auditory stimuli for 12 h per night for seven nights. We collected blood 5 days before the first exposure to auditory stimuli. At the end of the study, animals were killed by decapitation followed by double pithing, and blood and testes were harvested. As a result of logistical constraints, we were unable to test the noise-exposed and control groups simultaneously; therefore, to control for possible seasonal effects, we used two separate control groups [control 1 (*n* = 10) and control 2 (*n* = 9)], tested immediately before and after the experimental group (traffic noise; *n* = 10), respectively.

### Preparation of auditory stimuli

For the experimental playbacks, we used 10 recordings, each 3 min in duration, of different automobiles with energy up to 7 kHz (Lewis, 1973). We then used the pitch shift feature in Audition (Adobe v. 2.0) to shift each file ±1, 2 and 3 semitones, creating a total of 70 sound files. Control stimuli consisted of recordings of breeding choruses of captive animals from the same population from which the focal animals were taken. A total of 70 individual 3 min tracks of frog calls were generated; each track consisted of constant calling, i.e. no silence. A playlist was created on each of two iPod Nanos (Apple Corporation, Cupertino, CA, USA) with the 70 sound files (anthropogenic noise or chorus noise) and 70 3 min silent tracks. The iPods were then connected to their own powered amplifier (Pignose 7-100, Las Vegas, NV, USA) and set to shuffle. Experimental frogs were presented with recordings of both choruses of conspecifics and anthropogenic noise as in the study of Kaiser *et al*., (2011); control frogs were exposed only to conspecific playbacks. At any given time, a frog in the traffic noise treatment could be subjected to silence, chorus noise, anthropogenic noise or both noise types, mimicking the transience of these types of sounds in nature. All playbacks were broadcast at 70 dB SPL at 1 m (simulating a busy roadway from 10–30 m) for 12 h on each of seven consecutive nights, beginning at 20.00 h (i.e. from 1 h before lights-off in the animal room to 1 h before lights on). This interval was chosen to correspond to the putative activity periods for most calling amphibians. This population can call sporadically from dusk until early morning (B. Mailloux, personal communication).

### Blood collection

Blood was collected by non-terminal cardiac puncture 5 days before playbacks started and by exsanguination at the end of the experiment. Blood was centrifuged for 12 min at 13.3***g*** at 4°C, and plasma was stored at −80°C until assayed for CORT. All blood samples were collected between 10.30 and 11.30 h, when circulating CORT levels are near the circadian peak (K. Kaiser, K. McGovern, E. Wilson, and W. Saltzman, unpublished data).

### Corticosterone assay

Plasma corticosterone concentrations were determined in duplicate aliquots using a double-antibody ^125^I-radioimmunoassay kit (MP Biomedicals, Costa Mesa, CA, USA). We extracted samples in duplicate prior to assay by adding 500 µl methylene chloride and 500 µl ultrapure water (to aid in visualizing separation of phases) to 20 µl plasma, and allowed samples to incubate at room temperature for 30 (first extraction) or 15 min (second and third extractions) on an orbital shaker. After incubation, 90% of the methylene chloride was transferred to the assay tube. After the final extraction, assay tubes were dried in a water bath at 37°C under a stream of filtered air and then resuspended in 100 µl assay diluent. Tubes then were capped and stored at 4°C for 24 h before assay (Ouyang *et al.*, 2011). All other assay parameters were according to the manufacturer's instructions.

We evaluated parallelism and accuracy to assess the validity of the assay kit with *L. caerulea* plasma. Parallelism was determined by comparing the log–logit slope of three independent, serially diluted plasma pools, each containing plasma from at least two White's treefrogs, with that of the standard curve (Hoss *et al.*, 2011). Slope comparison was made using GraphPad Prism (v. 5.0; GraphPad Software, La Jolla, CA, USA). Slopes of pool dilutions were not distinguishable from the standard curve (*P* = 0.82). Accuracy was measured by adding known doses of CORT standards (*n* = 7, in duplicate) to two independent plasma pools, each containing plasma from at least 11 White's treefrogs. Recovery averaged 105% of expected.

All blood samples from the experiment were run in a single assay. Assay sensitivity was 60.2 pg/ml. The intra-assay coefficient of variation of an internal control plasma pool at the low end of the standard curve (89% bound) sampled in duplicate was 1.2%; the intra-assay coefficient of variation of an internal pool at the high end of the standard curve (28% bound) was 0.4%.

### Sperm count and viability

Testes were removed with microscissors and processed immediately. We minced testes with sharp microscissors in 5 ml 0.1× phosphate-buffered saline and incubated the mixture at room temperature for 60 min (R. A. Cardullo, personal communication). Sperm counts were measured as the average of two replicates counted on a haemocytometer. For analysis of sperm viability, we used a double-label method developed and validated in our laboratory for this species (K. Kaiser, K. McGovern, E. Wilson, and W. Saltzman, unpublished data). We labelled cells using CellTrace^™^ CFSE Cell Proliferation Kit (Invitrogen, Carlsbad, CA, USA) and with Fixable Viability Dye eFluor^®^ 660 (eBioscience, San Diego, CA, USA), both according to the manufacturer's instructions. CFSE labels all cells, whether living or dead; Fixable Viability Dye indicates only dead cells. Sperm were then fixed with paraformaldehyde and stored at 4°C for later analysis with a BD FACSCantoII flow cytometer (BD Biosciences, San Jose, CA, USA). We used count beads (Fluoresbrite Carboxylate microspheres; Polysciences, Warrington, PA, USA) to quantify the proportion of viable sperm in each sample. We counted 10 000 beads per sample and used FlowJo (v. 8.7.3; TreeStar, Ashland, OR, USA) for analysis.

### Statistical analysis

SPSS 21.0.0 (IBM-SPSS, Chicago, IL, USA) was used for analysis of CORT data. We used StataIC (v. 10 and v. 12; StataCorp, College Station, TX, USA) for all other statistical analyses. All data were tested for normality using the mvtest normality function in Stata v.12. Corticosterone data were log_10_ transformed and analysed using mixed-models general linear models with body mass as a covariate. Two outlier points (both from the control day –5 blood sample) were >3 SD from the mean and were excluded from the CORT analysis. Sperm data were log_10_ transformed and analysed by analysis of covariance (ANCOVA), with body mass at the end of the experiment included as a covariate.

## Results

**Figure 1:**
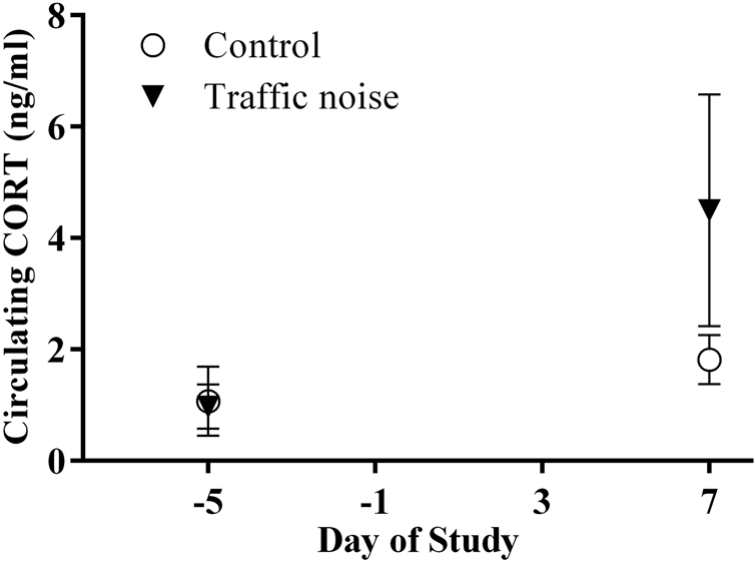
White's treefrogs in the traffic noise group (exposed to anthropogenic noise and natural chorus noise for seven nights; *n* = 10) had significantly increased plasma corticosterone (CORT) concentrations over those in the control group (exposed to only conspecific chorus noise for seven nights; *n* = 19). As expected, both groups showed increases in CORT concentrations at the end of the exposure period. Statistical analyses were performed on logarithmically transformed data; back-transformed data (mean values ± 95% confidence intervals) are presented.

Circulating concentrations of corticosterone did not differ between the control 1 and control 2 groups (treatment, *F*_1,5_ = 1.262, *P* = 0.312; treatment × day interaction, *F*_1,5_ = 4.659, *P* = 0.084; Table 1), so these groups were pooled into a single control group for further analyses. Frogs in the control and traffic noise groups had similar concentrations of plasma CORT at the beginning of the experiment (*F*_1,14_ = 0.401, *P* = 0.537). Circulating CORT concentrations increased both throughout the course of the experiment and with exposure to traffic noise (Fig. 1). As expected, both groups demonstrated an increase in plasma CORT concentrations over the course of the study (control, *F*_1,29.590_ = 14.299, *P* = 0.001; traffic noise, *F*_1,36.495_ = 26.798, *P* < 0.001), but frogs subjected to traffic noise had a higher mean circulating CORT concentration at the end of the experiment (*F*_1,23_ = 8.152, *P* = 0.009). The interaction between treatment and day was not significant (treatment, *F*_1,34.391_ = 5.992, *P* = 0.020; day, *F*_1,34.391_ = 40.918, *P* < 0.001; interaction, *F*_1,34.391_ = 2.376, *P* = 0.132). Circulating CORT concentrations did not covary with body mass at the end of the experiment (*F*_1,21_ = 2.75, *P* = 0.112). Although the observed CORT concentrations were low relative to other amphibian species, previous experiments have shown that this species has consistently low circulating CORT concentrations (both basal and stressed) across seasons (unpublished data; Peterson *et al.*, 2013).

**Table 1:** Mean, standard error and sample size for all measures, as well as average body mass at the end of the experiment, for each treatment group

Group	Circulating CORT, day −5 (pg/ml)	Circulating CORT, day 7 (pg/ml)	Sperm count (millions)	Sperm viability (% viable)	Average mass at end of experiment (g)
Control 1 (pre-control)	1668.6 ± 591.5 (*n* = 4)	2155.7 ± 659.0 (*n* = 9)	5.4 ± 1.3 (*n* = 10)	82.3 ± 1.0 (*n* = 5)	31.5
Control 2 (post-control)	1009.3 ± 347.6 (*n* = 7)	2060.5 ± 306.3 (*n* = 9)	5.7 ± 0.79 (*n* = 9)	86.9 ± 1.5 (*n* = 9)	28.2
Combined controls	1249.0 ± 307.2 (*n* = 11)	2108.1 ± 352.7 (*n* = 18)	5.5 ± 0.77 (*n* = 19)	85.2 ± 1.3 (*n* = 14)	29.9
Traffic noise	971.7 ± 167.4 (*n* = 8)	4100.8 ± 898.2 (*n* = 10)	2.4 ± 0.70 (*n* = 10)	63.7 ± 7.9 (*n* = 9)	35.1

**Figure 2:**
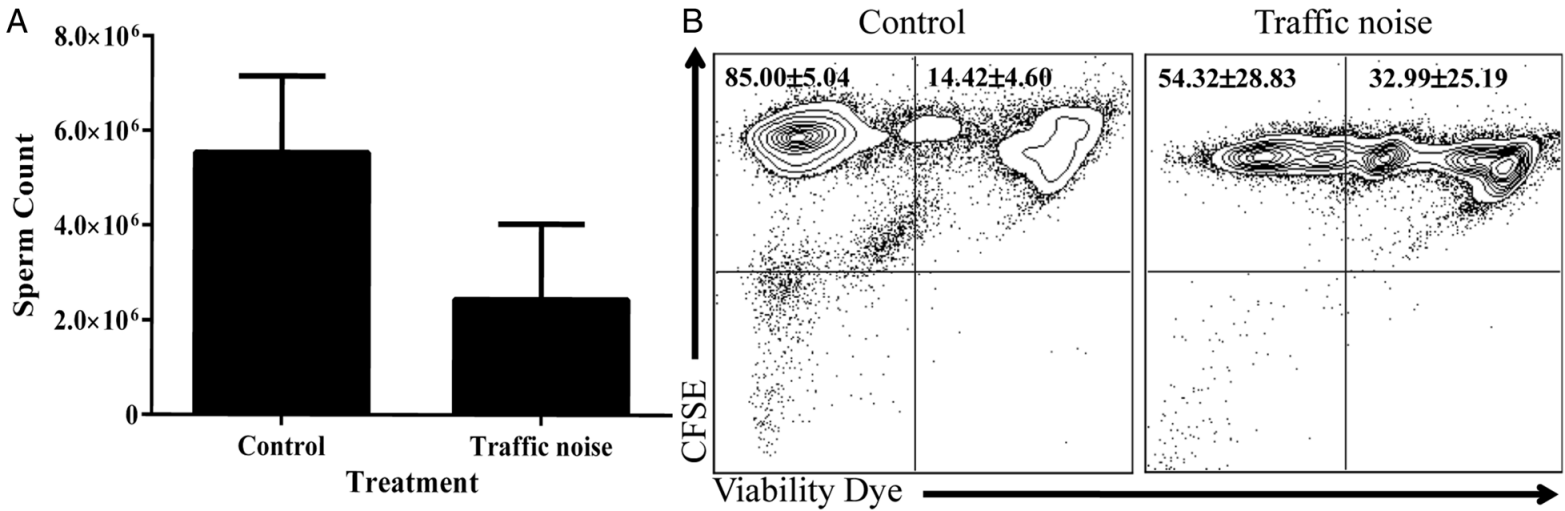
Traffic noise reduced sperm quantity and quality in White's treefrogs. (**A**) Frogs in the traffic noise group had significantly lower sperm counts than control frogs (*P* < 0.01, shown are means ± 95% confidence intervals). Analyses were conducted on logarithmically transformed data, which were then back transformed for presentation. (**B**) Frogs in the traffic noise group also had lower sperm viability (*P* < 0.01) at the end of the experiment relative to control frogs (*n* = 19). CFSE labels all cells, whereas viability dye labels only dead cells. More intense labelling with viability dye indicates an increase in dead cells. The plot shown is representative of all replicates. The mean proportions ± SD of live cells (upper left quadrant) and dead cells (upper right quadrant) are labelled for each group.

Raw data are presented for ease of interpretation, and data are presented separately for each of the three groups. For analyses and for Figs 1 and 2, the two control groups were pooled because there were no statistically significant differences between them; these pooled values are also shown. Note that data were logarithmically transformed for analyses, and the figures depict back-transformed data corrected for body mass (see main text for explanation). Abbreviation: CORT, corticosterone.

The control 1 and control 2 groups did not differ in sperm count (*F*_1,17_ = 0.560, *P* = 0.464; Table 1) or sperm viability (*F*_1,12_ = 3.576, *P* = 0.083), so animals in the two control treatments were pooled for analysis.

Compared with control frogs, frogs exposed to the traffic noise stressor showed significant depressions in both sperm count (*F*_2,26_ = 8.67, *P* = 0.007; Fig. 2A) and sperm viability (*F*_2,20_ = 6.93, *P* = 0.016; Fig. 2B). The interaction between body mass and treatment was not significant in either analysis (sperm count, *F*_1,25_ = 3.13, *P* = 0.089; sperm viability, *F*_1,19_ = 0.800, *P* = 0.383) and was dropped from the models.

## Discussion

As predicted, plasma CORT concentrations in frogs exposed only to conspecific choruses in this study increased slightly but significantly over the course of the experiment, consistent with social modulation of CORT shown by other researchers (Burmeister and Wilczynski, 2000). At the end of the noise exposure, however, frogs in the traffic noise treatment had significantly higher circulating CORT concentrations than did chorus-exposed control frogs, demonstrating a distinct effect beyond social mediation. Likewise, a recent study demonstrated that acute exposure to traffic noise increased circulating CORT concentrations in female *Lithobates sylvaticus* (Tennessen *et al.*, 2014). We also found that animals exposed to anthropogenic noise at ecologically relevant levels showed a significant decrease in sperm count and sperm viability compared with control frogs. Thus, our results add to the body of literature suggesting that noise associated with urbanization can be detrimental to vertebrates (reviewed by Barber *et al*., 2010; Slabbekoorn *et al.*, 2010; Halfwerk *et al.*, 2011; Kight and Swaddle, 2011).

The reduction in sperm count and viability was dramatic but not without precedent. Although we are not aware of any other work demonstrating such effects in amphibians, chronic stress has been linked to decreases in gamete count and quality in other vertebrate taxa (Campbell *et al.*, 1992, 1994; Almeida *et al.*, 2000; Schreck *et al.*, 2001). The mechanisms underpinning this decline have yet to be determined, but evidence that the reproductive hormone axis is impacted by stress across vertebrates is abundant in the literature (Rivier and Rivest, 1991; Wingfield and Sapolsky, 2003; Kirby *et al.*, 2009). Whether animals acclimatize to environmental stressors so that the observed effects on glucocorticoid concentrations and sperm function would dissipate over time remains to be tested, as does the effect on fitness in this system. If male *L. caerulea* exposed to chronic anthropogenic noise are able to sire the same number of viable offspring as other males, the observed decrease in sperm count and sperm viability may be trivial. However, because most frog species attract mates using acoustic advertisement calls, exogenous noise can interfere with the most important channel of communication among conspecifics and thus may impose a fitness cost outside of any stress-related costs.

In this study, frogs in the traffic noise group were exposed to anthropogenic noise in addition to chorus noise, so we did not control for total sound energy broadcast. We chose this design because in nature, traffic noise does not necessarily supplant conspecific and heterospecific noise. This design unfortunately makes it impossible to determine whether the increases in CORT and decreases in sperm were caused by traffic noise *per se* or simply by added sound energy. However, several species of frogs increase call rates in response to exogenous noise (Sun and Narins, 2005; Kaiser and Hammers, 2009; Cunnington and Fahrig, 2010). Such behavioural plasticity suggests that addition of traffic noise in a habitat may increase the total level of biotic sound as well. Thus, we suggest that even if our results were attributable simply to an increase in total sound noise, addition of exogenous noise is still likely to result in a stronger effect than increased natural calling alone.

Several caveats apply to our work. First, we used animals that were bred and housed in captivity. Baseline and stress-induced CORT concentrations are not necessarily similar between captive and wild animals (Calisi and Bentley, 2009). Furthermore, the relationship between stress and reproductive suppression may differ between wild and captive animals, although several established models suggest otherwise (Wingfield and Sapolsky, 2003; Romero *et al.*, 2009). Likewise, captive animals may have a different sensitivity to noise than do wild animals. Second, we did not determine the maximal CORT response to stress in this species; however, even if sub-maximal, the elevated CORT concentrations that resulted from noise exposure were associated with reductions in reproductive measures, whether or not these reductions were mediated by CORT. Finally, we measured CORT concentrations and sperm measures at only one time point following the onset of treatment. We used this design in order to reduce disturbance to the frogs to avoid confounding the effects of noise treatment. Even non-invasive sampling (e.g. urine sampling) requires handling of animals, which is likely to increase CORT concentrations; indeed, handling is a commonly used acute stressor (Moore *et al.*, 1991; Langkilde and Shine, 2006; Narayan *et al.*, 2012, 2013) and might therefore have obscured the response to traffic noise in this study. As a result, it is not clear at what point the observed increases in CORT and decreases in sperm count and sperm viability occurred.

Future studies will characterize the maximal CORT response to stress in White's treefrogs, the effects of noise on sperm count and viability over time, and whether these effects would ultimately lead to reductions in fitness. Myriad studies demonstrate that habitat alteration negatively affects amphibian populations through multiple mechanisms (e.g. Stuart *et al.*, 2004; Wake and Vredenburg, 2008). The rapidly changing landscape many individuals face as a result of habitat change may include a variety of potentially novel stressors, making it harder for animals to acclimatize to environmental disturbances. Studies incorporating multiple chronic environmental stressors would be valuable.

We therefore see the present study as an early contribution towards disentangling the complex effects of chronic anthropogenic stress and integrating them into a more comprehensive framework for developing conservation priorities. The successful synthesis of ecology, behaviour and physiology requires both fieldwork involving wild animals and laboratory studies in carefully controlled conditions (Calisi and Bentley, 2009). Effective integration of such studies can help us to understand and predict population- and community-level responses to anthropogenic change and species’ fates in the face of habitat loss and ever-increasing urbanization (Bonier, 2012; Fragkias *et al*., 2013).

## Funding

This work was supported by the National Science Foundation [Grant DBI-1003283 to K.K.].
